# Molecular characterization of atherosclerosis in HIV positive persons

**DOI:** 10.1038/s41598-021-82429-4

**Published:** 2021-02-05

**Authors:** Adam Cornwell, Rohith Palli, Meera V. Singh, Lauren Benoodt, Alicia Tyrell, Jun-ichi Abe, Giovanni Schifitto, Sanjay B. Maggirwar, Juilee Thakar

**Affiliations:** 1 Department of Biomedical Genetics, University of Rochester, Rochester, NY USA; 2 Medical Scientist Training Program, University of Rochester, Rochester, NY USA; 3 Biophysics, Structural, and Computational Biology PhD Program, University of Rochester, Rochester, NY USA; 4 Department of Microbiology and Immunology, University of Rochester, Rochester, NY USA; 5 Department of Neurology, General Neurology, University of Rochester, Rochester, NY USA; 6 Department of Imaging Sciences, University of Rochester, Rochester, NY USA; 7 Department of Cardiology-Research, Division of Internal Medicine, The University of Texas MD Anderson Cancer Center, Houston, TX USA; 8 Texas A&M Health Science Center Institute of Biosciences and Technology, Houston, TX USA; 9 Department of Microbiology, Immunology, and Tropical Medicine, George Washing University, Washington, DC USA; 10 Department of Biostatistics and Computational Biology, University of Rochester, 601 Elmwood Avenue, , Box 672, Rochester, NY 14642 USA

**Keywords:** Gene regulation, miRNAs, Network topology, HIV infections, Atherosclerosis

## Abstract

People living with HIV are at higher risk of atherosclerosis (AS). The pathogenesis of this risk is not fully understood. To assess the regulatory networks involved in AS we sequenced mRNA of the peripheral blood mononuclear cells (PBMCs) and measured cytokine and chemokine levels in the plasma of 13 persons living with HIV and 12 matched HIV-negative persons with and without AS. microRNAs (miRNAs) are known to play a role in HIV infection and may modulate gene regulation to drive AS. Hence, we further assessed miRNA expression in PBMCs of a subset of 12 HIV+ people with and without atherosclerosis. We identified 12 miRNAs differentially expressed between HIV+ AS+ and HIV+ , and validated 5 of those by RT-qPCR. While a few of these miRNAs have been implicated in HIV and atherosclerosis, others are novel. Integrating miRNA measurements with mRNA, we identified 27 target genes including SLC4A7, a critical sodium and bicarbonate transporter, that are potentially dysregulated during atherosclerosis. Additionally, we uncovered that levels of plasma cytokines were associated with transcription factor activity and miRNA expression in PBMCs. For example, BACH2 activity was associated with IL-1β, IL-15, and MIP-1α. IP10 and TNFα levels were associated with miR-124-3p. Finally, integration of all data types into a single network revealed increased importance of miRNAs in network regulation of the HIV+ group in contrast with increased importance of cytokines in the HIV+ AS+ group.

## Introduction

Studies of human immunology highlighting variability across individuals have revealed that variation in functional responses is largely determined by non-heritable factors, most likely by environmental exposures^1–3^. Viral infections such as human immunodeficiency virus type-1 (HIV) interfere with cellular targets and cause derangement of the immune response. Combination anti-retroviral therapy (cART) has rendered HIV infection as a chronic manageable disease. However, persons living with HIV remain at increased risk of co-morbidities despite cART; the most frequent of these is cardiovascular disease (CVD) driven by atherosclerosis (AS)^4–6^. It is likely that cART and HIV itself play a role in AS^7^. This observation was also reported in a study that assessed carotid artery thickening, a marker of AS, which was up to 24% higher in persons living with HIV compared with uninfected sex- and age-matched persons, and was comparable in cART-naïve persons and with virologic suppression on cART^8^. Moreover, a large retrospective analysis indicates that HIV infection status independently confers an odds ratio for acute myocardial infarction of ~ 1.93 (95% CI, 1.21–2.93) after adjusting for traditional risk factors such as age and hypertension^9^. Other more recent studies also support this observation^9–11^. Thus, persons living with HIV are at higher risk of AS even with ongoing cART therapy^8,12,13^. Moreover, elevated AS prevalence in HIV-1 elite controllers indicates that inflammation can be induced by the presence of HIV infection^14^.

AS is triggered by a pro-thrombotic inflammatory environment that attracts platelets and monocytes to injured endothelial cells. As monocytes migrate into the vessel wall, they differentiate into macrophages. Macrophages exert their phagocytic activity, engulfing lipids, and forming the characteristic foam cells. These cells release further chemoattractant molecules, maintaining a process that leads to plaque formation^15^. Several molecular mechanisms induced by HIV infection and cART therapy lead to persistent inflammation and contribute to development of AS^16–19^. Treatment with cART is associated with increased cholesterol levels, a risk factor for AS^20^. HIV infection itself leads to higher levels of oxidized low density lipoprotein (LDL)^21^ which increases endothelial expression of monocyte and T cell adhesion markers^22^. Oxidized LDL, cholesterol crystals, and HIV infection itself activate the macrophage/monocyte inflammasome, which drives inflammation through activation of cytokines such as IL-18 and IL-1β^23,24^.

Cytokines and other soluble secreted signaling molecules, such as chemokines, regulate inter- and intra-cellular signaling, thus driving pathogenesis of HIV or AS. A handful of such interactions are known: for example, IL-21 promotes STAT3-mediated expression of miR-29 which in turn disinhibits antiviral gene expression and resistance to HIV infection in CD4+ T-cells^25^. Stimulation of macrophages with a combination of IFN-γ and mildly-oxidized LDL induces miR-155 expression and results in higher levels of the MCP-1/CCL2 chemokine and further promotes inflammation through inhibition of the transcription factor BCL6^26^. However, cytokine-miRNA-mRNA networks in persons with HIV who also have atherosclerosis have yet to be fully characterized. Thus, further study of cytokines together with miRNA is warranted to understand the interaction between HIV and atherosclerosis.

Micro-RNAs (miRNAs or miRs) may be a major factor in the complex pathophysiology of AS^27–29^. miRNAs are short non-coding ribonucleotide molecules, typically 22 nt, that serve as critical regulators of gene expression across nearly all life stages and tissues. miRNA dysregulation has been linked to AS through effects on cholesterol metabolism, lipid uptake by macrophages, inflammation, and angiogenesis^30–32^. A number of miRNAs have been suggested as potential therapeutic targets and biomarkers for AS, as well as other diseases and environmental exposures^33–36^. Such miRNAs include miR-126-5p which has been shown to enhance the response of monocytes to lipopolysaccharide stimulation when levels are altered in persons with HIV infection^37^, miR-132 which is associated with CD4 + T-cell activation and increased HIV replication^38^, and let-7c, miR-34a, and miR-124a which modulate innate immune activity^39^, among others^40–43^. Of the miRNAs with altered expression in HIV infection, some have been studied as putative biomarkers of AS in HIV infection or have been previously associated with AS, including miR-210, miR-7, and miR-331^44^, miR-155 and miR-223^45^, miR-125a-5p and miR-139-5p^46^, miR-132^47^, and miR-126, miR-145, and let-7c^43^. How miRNAs are dysregulated in AS and chronic HIV infection, and the targets they modulate to act as effectors of inflammation in those contexts remains unclear.

Given the complex roles of miRNAs, cytokines, and signaling events, here we aim to use systems-level approaches to investigate dysregulated networks involved in development of AS in people living with HIV. Though atherosclerosis itself is a tissue-specific pathogenesis, chronic, systemic inflammation (which is a hallmark of HIV infection) is one of the major drivers of AS progression^48,49^. In that regard, PBMCs provide insights into systems-level changes and are frequently used as a proxy to investigate tissue- and organ-specific modulations^50^. Specific to HIV infection, various components of anti-retroviral therapy and viral proteins exert inflammatory/activatory effects on monocytes/macrophages as shown by our group^7^ and others (reviewed in^51^), which are important cellular mediators of AS progression^52,53^. Further, miRNAs are often released into the blood in stable protein complexes or exosomes and have been implicated in patho-mechanisms of AS^54^. They can be potentially used to develop diagnostic tests and, in that respect, it is important that the acquisition of blood is low risk to individuals. To this end, we collected blood samples from 13 persons living with HIV and 12 uninfected persons, with and without AS. The collected blood was used to obtain multiple measurements of immune phenotypes including mRNA expression and soluble cytokine and chemokine abundance in all 25 subjects, in addition to miRNA expression in the subjects living with HIV. We find regulatory modes driven by cytokines and miRNAs play differential roles in HIV+ and HIV+ AS+ .

## Methods

### Participant cohort summary, sample collection, and storage

13 persons living with HIV and ≥ 50 years of age on stable cART for at least 1 year and with viral load ≤ 50 copies/mL were recruited. 12 HIV-negative persons matched for age, gender, environment and Reynolds CVD risk score were also recruited. The details of HIV treatment are available in Supplemental Table 1. All methods were carried out in accordance with University of Rochester guidelines and regulations, and all experimental and study protocols were approved by the University of Rochester Institutional Review Board (#RSRB00063845). Informed consent was obtained from all subjects. Individuals were assigned AS+ if they had carotid plaques on both sides, and AS− if they did not have carotid plaques. 30 mLs of blood per study participant was collected in ACD vacutainers and was processed within 2–3 h of collection. Peripheral Blood Mononuclear Cells (PBMCs) were isolated using Ficoll density gradient centrifugation. Five mLs of plasma was stored at -80ºC and 5 million PBMCs were preserved using RNAlater (Thermo Fisher). De-identified subject information is available in Supplemental Table 1.

### Levels of pro- and anti-inflammatory mediators in peripheral blood plasma

A 29-Plex Milliplex Human Cytokine/Chemokine panel kit (Millipore Cat# HCYTMAG-60K-PX29) was used to quantitate the cytokine and chemokine levels using Luminex magnetic microbead array technology. The assay was performed according to the manufacturer’s instructions. In brief, plasma specimens were thawed on ice and micro-centrifuged for 5 min at 15,000 RCF at 4 °C to remove any particulate matter. 25 µLs of reference standard dilutions, control, and test specimens were incubated overnight at 4 °C with shaking at 800 RPM in duplicate wells on a 96 well plate with 25 µLs of either assay buffer for specimens or plasma matrix for standards and controls and 25 µLs of microbead solution. Wells were washed 3 times with wash buffer on an automated magnetic plate washer (Bio-Rad Bio-Plex ProII) and 25 µLs of biotinylated detection antibodies were added for 1 h at room temperature (RT) with shaking. 25 µLs of Streptavidin–Phycoerythrin was then added per well and incubated for 30 min at RT before washing the plate 3 times. Finally, 150 µLs of phosphate-buffered saline at pH 7.2 was added per well and mixed prior to reading on a Luminex 200 instrument. Control 1 and Control 2 values for all analytes fell within the kit quality control ranges for the kit lot per the standard reference curve (range 3.2 pg/mL to 2000 pg/mL), confirming acceptable performance of the kit. The trimmed means of the fluorescence intensities across the beads for a given sample and target were exported from the Luminex Xponent software. The geometric mean of two technical replicates for each sample were used in downstream analyses. The data for the cytokine/chemokine panel is available in Supplemental Table 2.

### Peripheral blood mononuclear cell isolation and RNA-sequencing

mRNA and micro-RNA were isolated and sequenced from PBMCs at the UR Genomics Research Center using their standard protocols. mRNA libraries were prepared with the Illumina TruSeq Kit v2, using oligo-dT beads for poly-A selection. mRNA was sequenced as single-ended 100 bp reads, yielding an average of 26.7 million reads per sample, for a total of 25 samples, representing four sample groups (normal control, n = 6; AS+, n = 6; HIV+, n = 6; HIV+ AS+, n = 7). Reads were trimmed with Trimmomatic to remove low-quality read tails or entire reads if the remaining post-trim read length was too short. Genome mapping was performed with STAR 2.5.2b to Human genome GRCh38.p7 with annotation from Gencode Genes version 25. Read counting was performed with the featureCounts from Subread. Count data is available in Supplemental Table 3. Small RNA was size-selected from total RNA and libraries were prepared with the Illumina TruSeq Small RNA kit. Small RNA was sequenced as single-ended 50 bp reads, with an average of 17 million reads per sample, for a total of 12 samples representing two sample groups (n = 6 each for HIV+ and HIV+ AS+). Reads were trimmed, mapped, annotated, and counted with the miRge pipeline^55^. Count data is available in Supplemental Table 4. miRNA were mapped to cell types using an available catalogue of miR expression in blood cell types^56^.

### RNA-sequencing analysis

Small RNA-seq and mRNA-seq datasets were read into R (version 3.5.1)^57^, and filtered to remove sequences with low expression based on raw counts. For mRNA, any gene with fewer than 10 reads in all samples was excluded, leaving 19,861 genes for annotated gene types which may be polyadenylated (protein coding, lincRNA, and processed pseudogenes). For miRNA, any miRNA with fewer than 10 reads in all samples was excluded, yielding a set of 778 miRNAs used for downstream analyses. Exploratory analysis of each dataset (e.g. clustering, expression heatmaps, PCA) was performed on variance-stabilized transformed (VST) counts, as computed by the DESeq2 package (version 1.22.2)^58^. Functional and pathway enrichment was investigated with gProfileR (version 0.6.7)^59^. Differential expression analysis was performed with DESeq2^58^ for both mRNA and miRNA (independently). The results of differential gene expression analysis for mRNA for all genes in the dataset are available in Supplemental Table 5, and differentially expressed miRNAs are available in Supplemental Table 6. For mRNA, groupwise comparisons were performed a corrected (FDR) p-value and fold-change cutoffs are noted in the results. Predicted miRNA target genes were found for differentially expressed miRNAs with the miRNAtap package (version 1.16.0)^60^ which incorporates predictions from five algorithms: DIANA, Miranda, Targetscan, PicTar, and miRDB. A given miR-target relationship was recorded if it was supported by at least two of the five prediction algorithms. The predicted targets of differentially expressed miRNAs are available in Supplemental Table 7, while functional enrichment analysis on these targets is available in Supplemental Table 8.

### miRNA expression across normal human blood cells

Publicly available miRNA expression data across normal human blood cells were downloaded from GEO (GSE100467)^56^, and read into R with the BioConductor package IsoMirs (version 1.10.1)^61^ to obtain read counts for each of the 450 samples, representing ten cell populations from 162 unique donors. A matrix of raw counts was assembled by joining the individual sample results with dplyr, and miRNAs with very low counts (< 3) across all samples were excluded, leaving 1062 unique miRNAs with expression in at least one sample. Variance-stabilized (VST) and library-normalized counts were obtained with DESeq2. To obtain a summary of expression of each miRNA for each cell type, miRNA abundances were discretized in 5 levels, with higher-value bins representing higher expression. The number of bins was determined by $ceiling\left(\sqrt[3]{max\left(n\right)}\right)$, where $n$ is the number of samples for each cell type^62^. Relative levels of miRNA expression in each cell-type was given by the median of the discretized values.

### Data integration analysis

Three types of data integration were performed; (1) miRNA and mRNA (2) cytokine and mRNA and (3) cytokine, mRNA and miRNA datasets were combined. Predicted miRNA targets were further filtered based on their significant negative correlation with mRNA (p < 0.05, $r\le -0.5$). Linear discriminant analysis (LDA) was performed on predicted miRNA target genes to identify genes that distinguish between HIV+ AS− and HIV+ AS+ samples. To identify transcriptional changes associated with cytokine levels, a single person transcription factor score (spTFscore) was calculated. Particularly, transcription factor (TF) target genes were predicted using positional weight matrices from JASPAR^63^ as described^64,65^. To calculate spTFscore, PCA was performed on TF target genes and $\sqrt{{\sum }_{i=2}^{m}{s}_{i}^{2}*{v}_{i}}$ was calculated from $i=2$ to $m$, the PC at which 50% cumulative variance is explained (including PC_1_), $s$ is the unweighted PC score, and $v$ is the % variance explained by the PC. Since PC_1_ was associated with inter-individual variability in overall transcription levels rather than inter-group and transcription factor specific variability, the score was calculated starting with PC_2_. Thus, the spTFscore captures the contribution of each TF measured by variance of their target gene expression in each person. Pearson correlation was measured between all plasma cytokines and spTFscore. TF target gene weights were calculated as contributions to spTFscore by $\frac{\sqrt{{\sum }_{i=2}^{m}{w}_{i}^{2}*{v}_{i}}}{1-{v}_{1}}$ where ${w}_{i}$ is the loading of the gene in that PC, $v$ is the variance of each PC,  ${v}_{1}$ is the variance explained by the first PC and $m$ is the PC at which 50% cumulative variance is explained, as with the spTFscore. In this way, the gene TF weights reflect the degree to which each gene contributes to the spTFscore for that TF.

Finally, to integrate miRNA, mRNA, and cytokine datasets an integrated multi-omic network was constructed and differential network analysis performed using xMWAS^66^. In order to include all data types, only samples from HIV+ AS− and HIV+ AS+ groups were included. Briefly, xMWAS constructs a matrix for pairwise correlation analysis using sparse partial least squares then ranks and filters the top association scores by p-value and association score. The edge lists are merged to a global network upon which community detection algorithms are utilized to group nodes into communities. Further, centrality scores are calculated for each node in each group separately, then compared to generate differences between groups. These centrality scores are available in Supplemental Table 9. Graphical representations of these networks were constructed in Cytoscape (version 3.7.0)^67^.

### Validation of miRNAs

In order to validate RNA-sequencing results, levels of miRNAs 144-3p, 144-5p, 183-5p, 451a, and 4732-3p were measured in a subset of samples used for RNA-seq (n = 2 HIV+ AS+ , and n = 2, HIV+ AS−). In addition, we also measured the expression of these miRNAs in new samples (not used in RNA-seq, n = 3 HIV+ AS+ and n = 3 HIV+ AS−). RNA from new samples was extracted using miRNeasy mini kit (Cat # 217004, Qiagen). RT-qPCR was performed as per the manufacturer’s protocol (Taqman microRNA assay kits, Cat # 4427975 and 4440886) using the Biorad CFX Connect real time PCR machine. miRNA U6 was used as an endogenous control. The data is shown as ΔCt values. The results were analyzed by unpaired t-test using Graphpad Prism software.

## Results and discussion:

### Gene signatures of AS in persons living with HIV

Differential expression analysis identified seventeen genes in each of the two comparisons, HIV vs HIV+ AS+ and HIV vs control group (p-adj < 0.05). There was one common genes among the two contrasts, integrin alpha D (ITGAD/CD11d), which is strongly associated with inflammation^68,69^. Comparison between the HIV+ AS+ and control groups revealed five genes higher in HIV+ AS+ group: CD8A, CD8B, NPDC1, JAKMIP1, and STYK1; with five higher in control: IL17RE, SPTSSB, TPBG, OVOS, COL13A1. The presence of CD8A and CD8B provides strong evidence that there is an increase in CD8 cell activity in HIV+ AS+ group. Previous reports have shown memory CX3CR1+ CD8(+) cells may contribute to atherosclerosis in persons living with HIV^70^. On the other hand, downregulation of the IL-17 receptor (IL17RE) could be in response to increased immune stimulation. However, large variation in gene expression across individuals within groups led to identification of few differentially expressed (DE) genes.

**Figure 1:**
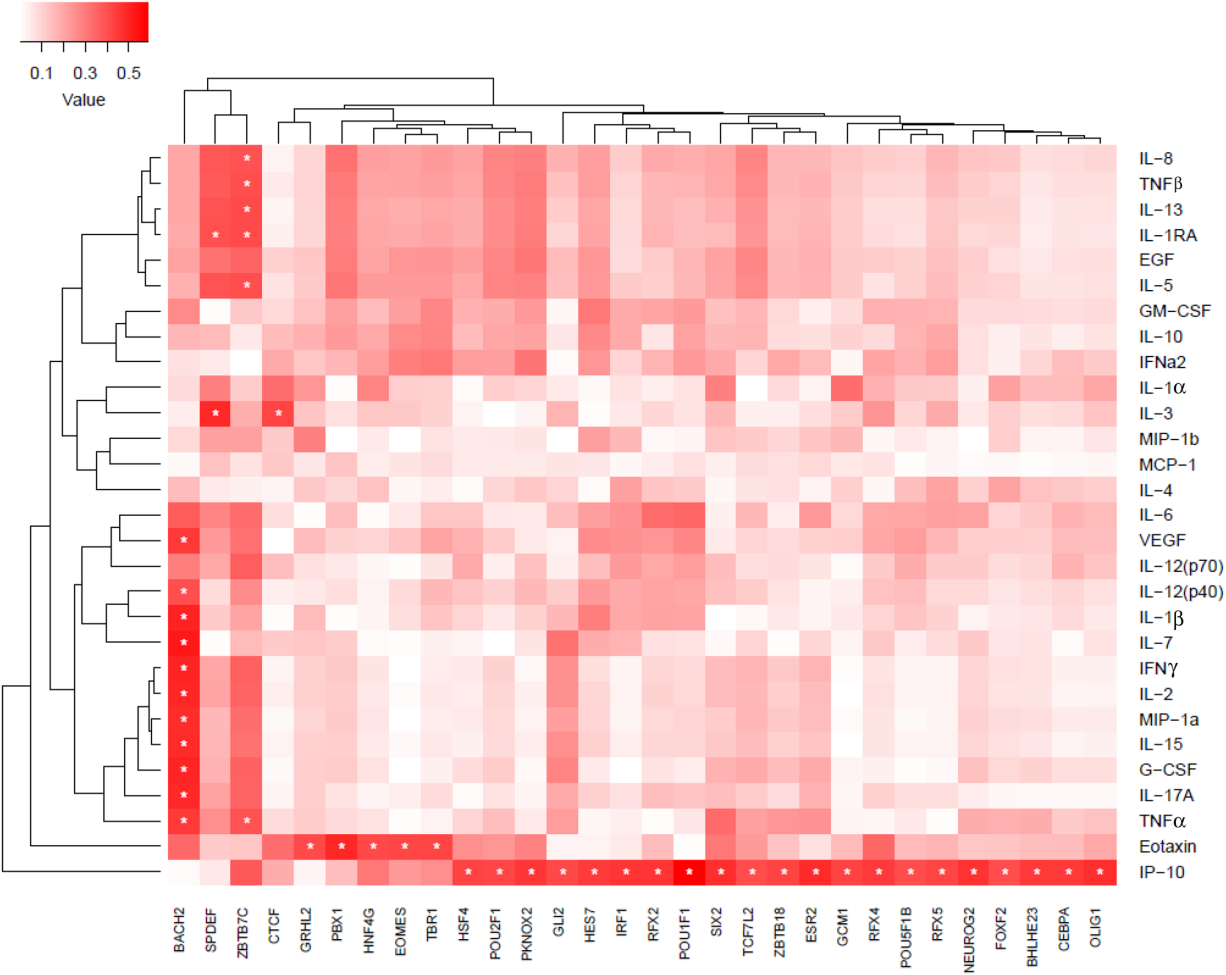
Association of activity of transcription factors (TFs) with cytokine and chemokine levels in plasma. Activity of the TFs was measured by single person transcription factor (spTF) scores. spTF was calculated as described in the methods. Briefly, TF targets as described in^64^ were used to perform PCA and subject specific PC scores were used to calculate pearson correlation (ranging from low to high depicted by white to red color bar) with cytokine levels. The significant correlations (p < 0.05) are indicated with stars. Analysis and graphical output done using in R (ver 3.5.1)^57^.

To investigate transcriptional changes in this highly variable data, we developed a single person transcription factor score (spTFscore) that could be compared with cytokine levels to identify transcription factors regulated by cytokines. The correlations between cytokine levels and the spTFscore revealed regulation by different arms of the immune system. Specifically, Th1, Th17 signature cytokines along with TNFα, MIP-1α and GCSF were associated with BACH2. It is known that Bach2–Batf interactions are required to prevent an excessive Th2 response^71^. IP10 was associated with several transcription factors such as IRF1  and CEBPα. Further analysis indicated that predicted targets of these transcription factors have little overlap, suggesting diverse transcriptional programs potentially associated with IP-10. IL-13, IL-8, TNFα, IL-1R and GMCSF were all associated with ZBTB7C, a TF that represses matrix metalloproteinases (MMPs) (Fig. 1). A number of the cytokines in this list are important modulators of atherosclerosis, including IL-15 which stimulates T cells and is known to be expressed in atherosclerotic lesions^72–75^.

**Figure 2:**
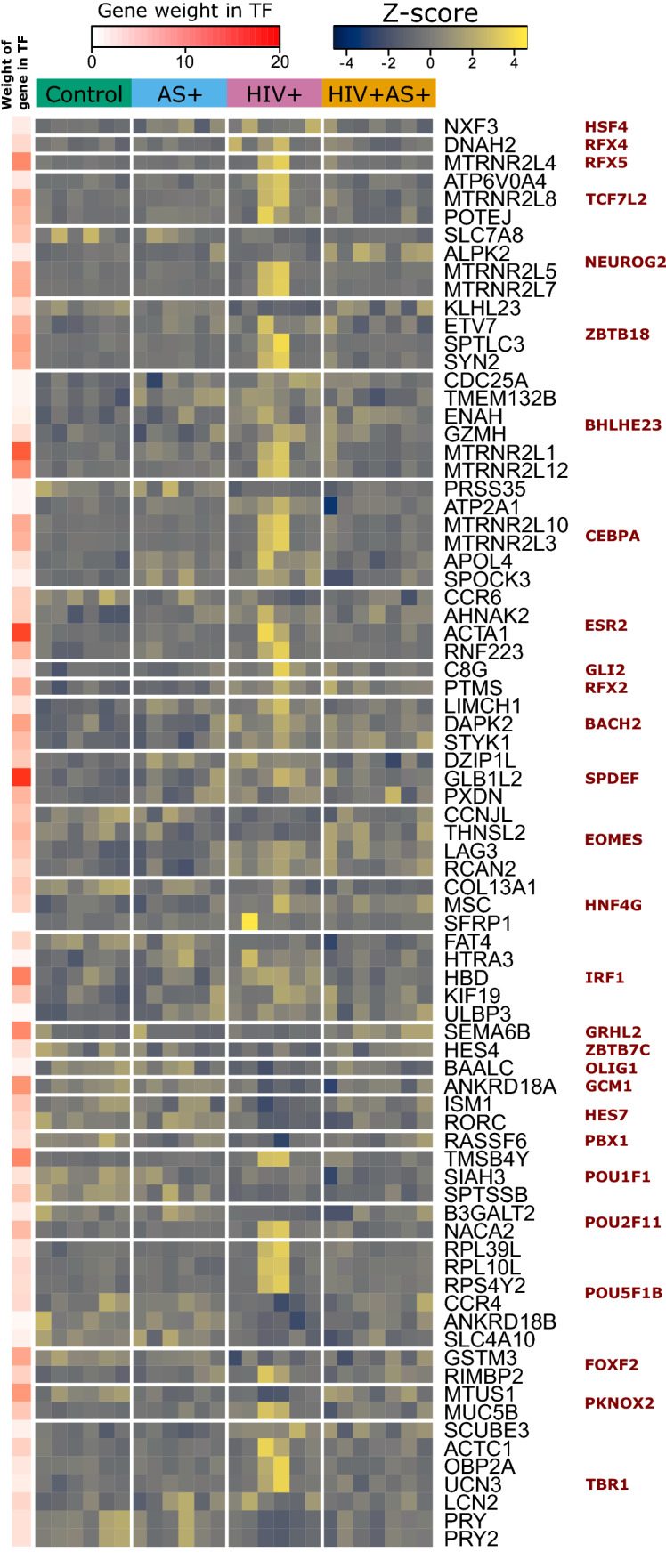
Gene targets of transcription factors correlated $|r^2|>=0.4$ with cytokine and chemokine levels in plasma. Expression of the differentially expressed targets (p-adj < 0.1, LFC > 1.0 in any comparison) of transcription factors from Fig. 1. Expression shown as z-scores across all four groups (indicated by top bar). The weight of each gene in determining spTF score is shown on left (white to red color bar) as the percentage of the variance accounted by the gene. Analysis and graphical output done in R (ver 3.5.1)^57^.

Many target genes of TFs significantly associated with the cytokines were differentially expressed (p-adj < 0.1 and LFC > 1.0) between HIV+ vs HIV+ AS+ group and HIV+ vs healthy (Fig. 2). The genes differentially expressed between HIV+ vs healthy and targets of TFs associated with IP10 include genes implicated in HIV infection e.g. CCR4, CCR6, and KIF19. CCR4 + and CCR6 + CD4 + T cells are highly permissive to HIV replication^76^. Interestingly, IP-10 enhances the recruitment of HIV-target cells and is a biomarker of early disease onset^77^. Eotaxin, a cytokine that was significantly different between HIV+ and control groups was also associated with several TFs, including GRHL12, PBX1, EOMES, and TBR1. Levels of circulating LAG3, an Eomes target gene, have been associated with HDL cholesterol and risk of coronary artery disease^78^. LAG3 here had increased expression in HIV+ group compared to control.

We also evaluated additional genes different at p-adj < 0.1 and LFC > 1.0 and associated with either HIV infection or AS (Supplementary Table 5). ATP1A2 is an Na^+^/K^+^ pump component involved in smooth muscle contraction, and it is differentially expressed in vasculature between myocardial infarction (MI) and stable angina^79^, and is also differentially expressed in our data between HIV+ and HIV+ AS+ . SLIT2 is a known inhibitor of HIV transmission^80^ that was significantly elevated in HIV+ vs control. GPR15 is a co-receptor for HIV and a possible biomarker for smoking status^81,82^; it had increased expression in HIV+ AS+ than HIV+ group. In this context, it may be a marker for the deleterious interaction between HIV and smoking status, which leads to atherosclerosis. VCAM1, a critical pro-atherosclerotic factor expressed on the endothelium, was upregulated in HIV+ vs control.

In conclusion, transcriptional changes and their relation with cytokine levels reveals several dysregulated interactions contributing to AS in the context of HIV and suggests further risk in HIV+ persons.

### Characterization of micro-RNAs in HIV+ persons with carotid plaques

**Figure 3:**
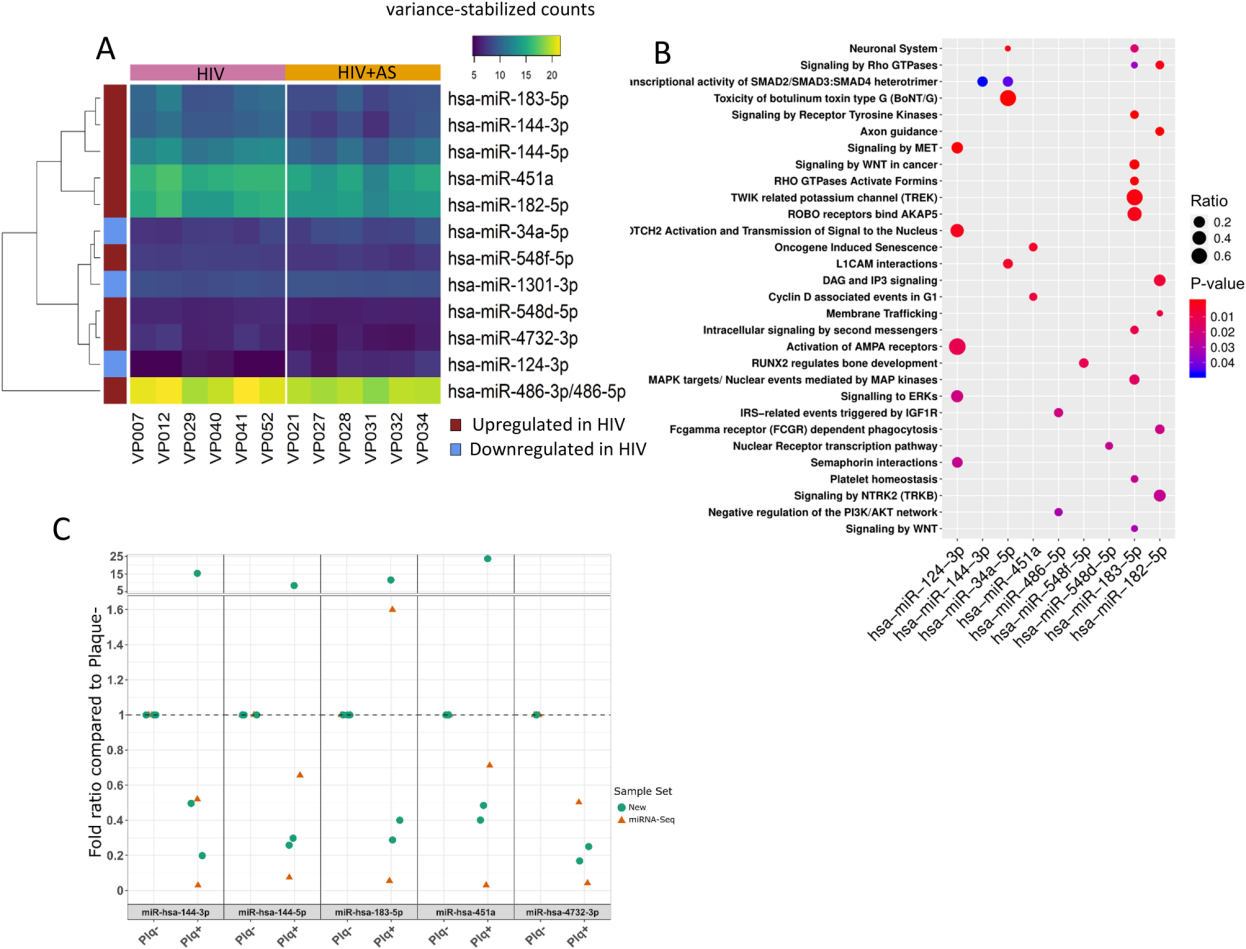
Dysregulation of miRNAs during atherosclerosis in HIV positive persons: **(A)** Differentially expressed miRNA counts. HIV+ samples shown on left (pink bar on the top) and HIV+ AS+ on right (orange bar on the top). miRNAs are clustered hierarchically with directionality shown by a left red (high in HIV+ group)/blue (high in HIV+ AS+ group) color bar^56^. **(B)** Pathway analysis of predicted target genes of DE miRNA. Dots represent significantly enriched pathways with color representing p-value and size of dot representing proportion of target genes in the pathway. **(C)** Fold-ratio of selected miRNAs in HIV+ AS− (Plq-) and HIV+ AS+ (Plq +) samples, as measured by RT-qPCR. Samples included a subset of those previously profiled by miRNA-Seq (orange triangles), as well as new samples (green circles). Graphical output for **(A–C)** and analysis for **(A,B)** done in R (version 3.5.1).

We performed genome-wide miRNA sequencing in HIV+ persons with (n = 6) and without (n = 6) carotid plaques. All 12 participants were males of ≥ 50 age to minimize the inter-person variation and maximize the differences across disease/infection groups in this small study. Moreover, in this focused group confounding variables were balanced, increasing the statistical power. Sequencing yielded at least 15 million short reads per sample, and most reads were ~ 22 nt after trimming, the typical length of miRNAs, indicating successful size selection and good read quality. Less than 10% of reads remained unmapped in all cases. miRNAs were identified and quantified from sequencing data with the miRge pipeline^55^. 778 miRNAs were considered after filtering out miRNAs that did not have at least 10 reads in all samples. Differential expression analysis identified 12 microRNAs with significant expression changes, out of which 3 were higher in HIV+ subjects with AS and 9 were higher in HIV+ subjects without AS (Fig. 3A).

A subset of these differently expressed miRNAs that have been previously implicated in cardiovascular disease are discussed below. High miR-124-3p indicated susceptibility to atherosclerosis in a population of smokers^83^. In our dataset, miR-124-3p had no or very low expression in HIV+ AS− samples, but was found in 5 of 6 HIV+ AS+ samples (only two of which were identified as current smokers) indicating that the elevated level of miR-124-3p may be indicative of AS risk outside of the context of smoking. Another miRNA, miR-182-5p is found to be upregulated about two times in myocardial infarction vs controls in whole blood samples, however this miRNA was downregulated in HIV+ persons with AS compared to without^84^. miR-34a-5p is expressed in T-cells (CD3+ and CD4+), and is negatively correlated with T-cell counts after HIV infection, possibly due to lysis of T-cells^85,86^. miR-34a-5p has also been shown to be increased in heart failure and decreased in peripheral arterial disease^87–89^, in addition to being upregulated in endothelial progenitor cells from coronary artery disease patients^90^. In ApoE-deficient mice fed a “western-style” diet for 16 weeks, the mice developed severe atherosclerotic lesions and exhibited progressive upregulation of miR-34a-5p^91^. In accordance with these observations linking higher levels of miR-34a-5p to AS, we find this miRNA to be elevated in most of our HIV+ AS+ samples compared to HIV+ alone.

To validate the miRNAs identified by RNA-seq we performed RT-qPCR on 5 miRNAs in ten participants (Fig. 3C). The miRNAs miR-144-3p, miR-144-5p, miR-183-5p, miR-451a and miR-4732-3p were chosen based on the largest fold change between HIV+ AS+ vs HIV+ AS−. The samples were run in pairs as shown in Fig. 3C. All of the 5 miRNAs were lower in HIV+ AS+ compared to HIV+ AS− in RNA-seq experiments. RT-qPCR validated this observation when a subset of the same samples from RNA-seq were used. Among the new independent samples one pair showed an opposite direction, whereas all other samples validated the observations from RNA-seq. The five validated miRNAs are novel and very little is known with respect to their role in HIV infection and atherosclerosis. miR-144-3p^92,93^ and miR-183-5p^94^ have been linked atherosclerosis, cholesterol metabolism, and HIV infection in different organisms and model systems. In consensus with our observations miR-451a has been found to be reduced in circulation of subjects with coronary heart disease^95^.

To investigate which specific cell-types are known to express the differentially expressed miRNAs from our study, we used a public compendium of miRNA expression across human blood cell types^56^. Specifically, expression of differentially expressed miRNAs was investigated in the public compendium to determine the cell types that normally express the differentially expressed miRNAs (Supplemental Fig. 1). This analysis revealed that only miR-486-5p was ubiquitously expressed, and that the other DE miRNA may exhibit low or no expression in normal CD8+ or CD4+ T cells and CD14+ monocytes (Supplemental Fig. 1). To further characterize the effect of these miRNAs, we performed pathway analysis of their predicted targets (Fig. 3B). Importantly, similar to previous findings, miR-124-3p was enriched in ‘Signaling to ERKs’ and ‘Activation of AMPA Receptors’. miR-183-5p was associated with a number of pathways including the ‘Twik related potassium channel (TREK)’, ‘Signaling by Receptor Tyrosine Kinases’, and ‘ROBO receptors bind AKAP5′. Finally, miR-182-5p was associated with ‘DAG and IP3 Signaling’, ‘Signaling by Rho GTPases’, and ‘FCγ receptor (FCGR) dependent phagocytosis’^96,97^.

Thus, most of our findings corroborate with previously known roles of miRNAs in atherosclerosis, and reveal novel insights about development of AS in persons living with HIV. However, most previous studies have been performed with different model systems, experimental designs, and protocols, necessitating extended study in people living with HIV.

### Role of miRNAs in regulatory networks

**Figure 4:**
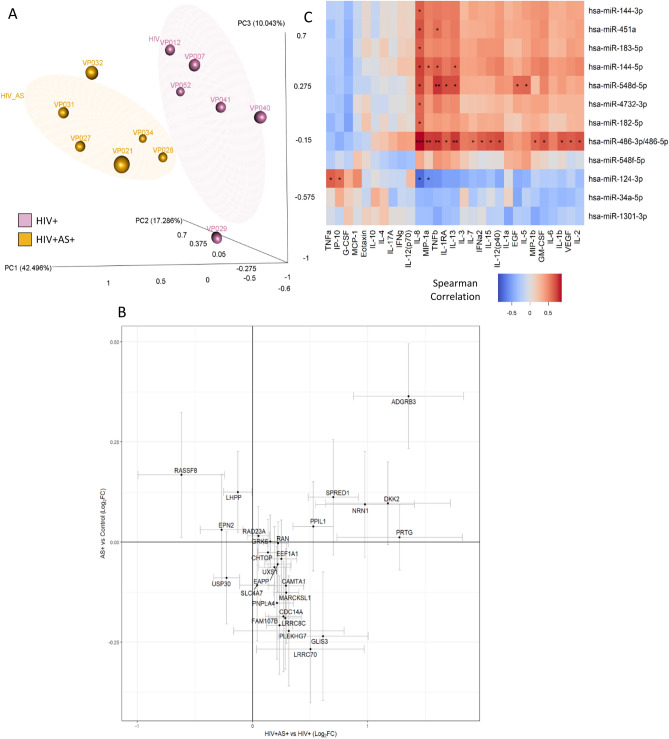
Interaction of miRNA expressed in PBMCs with mRNA expression in PBMCs and cytokines and chemokines levels in plasma: **(A)** miRNA target genes were identified using miRNAtap package^60^ and were further filtered to keep only genes whose expression was negatively correlated with the expression of their regulator miRNA. The filtered genes were analyzed by Linear Discriminant Analysis (LDA) and 27 target genes were selected based on separation between AS+ (yellow)/− (purple) groups. PCA with 27 genes are shown (s). **(B)** Log2 fold change of HIV+ AS+ vs HIV+ and AS+ vs healthy control for 27 target genes. **(C)** Association of miRNA with cytokine and chemokines using the data from HIV+ AS+ and HIV+ AS− groups. Spearman correlation; significant associations are indicated with stars (*p-value < 0.05, **p-value < 0.005, ***p-value < 0.001). Analysis and graphical output done in R (ver 3.5.1)^57^.

miRNAs are important regulators of mRNA turnover and are thought to regulate 10–30% of mRNAs^98–100^. miRNAs function post-transcriptionally via the RNA-induced silencing complex (RISC) to inhibit mRNA. Hence, we found predicted targets of differentially expressed miRNAs that also had significant negative correlation with mRNA expression in our data. A set of 27 of these genes with highest loadings from Linear Discriminant Analysis (LDA) was sufficient to differentiate the HIV+ AS+ and HIV+ AS− groups (Fig. 4A). This list included genes such as SPRED1 which is involved in IL-15 and FGFR1 signaling, GRK6 which is involved in the myometrial relaxation and contraction pathway and calcium regulation in cardiac cells, and RAN which is involved in the export of viral ribonucleoprotein. Also in this set of 27 genes is SLC4A7, a critical sodium and bicarbonate transporter involved in generation of Nitric Oxide (NO) signaling and blood pressure regulation; NO signaling has been linked to AS risk^101^. Importantly, 15/27 of these genes are upregulated in HIV+ AS+ vs HIV but downregulated in AS vs healthy control with no correlation between the two changes, indicating that these genes describe an HIV-specific atherosclerosis state (Fig. 4B).

miRNAs can be transcriptionally regulated by cytokine signaling^102^ while cytokine production^103^ and signal transduction can be influenced by miRNAs^104,105^. Hence, we asked which cytokines are significantly associated with DE miRNAs. IL-8 was positively associated with most of the miRNAs but negatively associated with miR-124-3p (Fig. 4C). miR-124-3p, which regulates ERK signaling and activation of AMPA receptors, was also negatively associated with MIP-1α but positively associated with TNFα and IP10 (also known as CXCL10). IP10 is induced during antiviral response and was higher in HIV+ and HIV+ AS+ groups (not statistically significant, p = 0.08). Numerous studies have reported abnormally high plasma IP-10 levels in the context of HIV infection, and IP10 is considered an important pro-inflammatory factor in the HIV disease process^106^. IP-10 and TNFα are both associated with miR-124-3p (Fig. 4C) and are highly expressed in HIV+ AS+ group. As discussed before, we found IP10 to be associated with diverse transcriptional programs activating several genes implicated in HIV infection. The role of miR-124-3p in this regulation needs to be further investigated. IL8 has interactions with miRNA in a number of diseases and conditions^107^. Other miRNAs have been found to de-repress expression of IL6 and IL8 in a cell-based model of sepsis^108^. These results suggest novel regulatory relationships between miRNAs and IL-8; however, IL-8 levels were not different in HIV+ persons with and without AS. IL-8 expression (at both the gene and plasma levels) has been reported to be significantly elevated in HIV-infected children that were not yet receiving ART therapy, and decreased after therapy began^109^. Several additional associations were found between miRNAs and cytokines (Fig. 4C). Thus, this work suggests a role for pro-inflammatory cytokines in regulating miRNAs which can in turn regulate gene programs.

### Integrated network of cytokines, miRNA and mRNA

**Figure 5:**
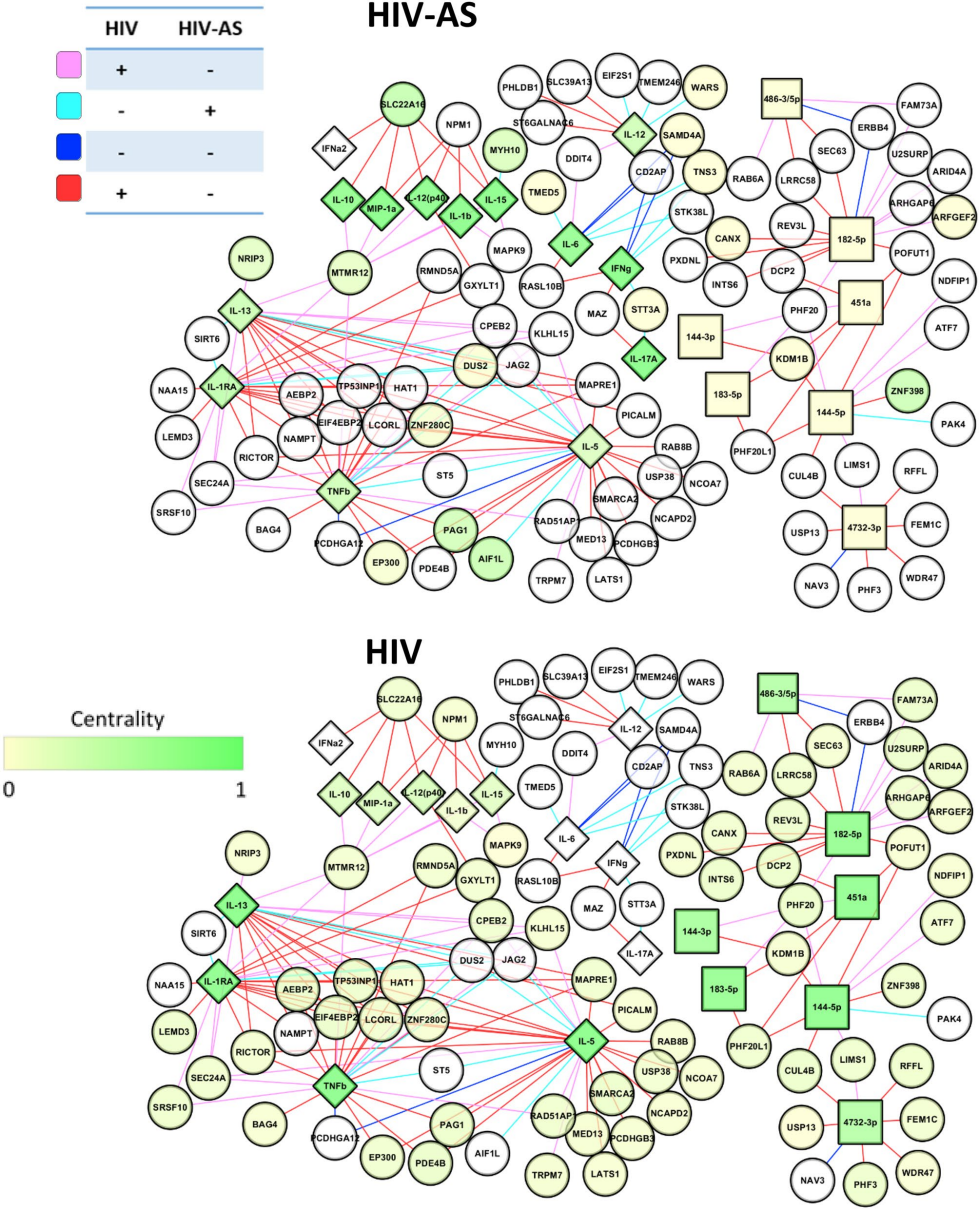
Regulation of AS associated signaling in the context of HIV. The network of association between miRNA, mRNA and cytokines in HIV+ people with and without AS. Squares represent miRNA, circles represent mRNA and diamonds represent cytokines and chemokines. Coloring of nodes indicates centrality score for HIV+ AS+ group (top) or HIV group (bottom). Coloring of edges represents sign of correlation between nodes in HIV+ or HIV+ AS+ groups as shown in the edge color key. Analysis performed in R (ver 3.5.1)^57^, with graphical output from Cytoscape (version 3.7.0)^67^.

Finally, having established several links between cytokines, miRNAs, and mRNAs in a pairwise manner, we set out to investigate such interactions systematically. Hence, we constructed a network, incorporating all three data types- mRNA, miRNA, and cytokine profiles, for HIV+ and HIV+ AS+ groups. The network includes 175 genes, 23 cytokines and 10 miRNAs (Fig. 5, Supplementary Table 1). Differential network analysis using eigenvector centrality identify nodes with higher importance with AS in the context of HIV.

Higher centrality of cytokines and chemokines compared to the genes was expected because a small number of cytokines measured were connected to many miRNA and genes. Three cytokines, IL1β, IL15, and MIP1α, which were also associated with BACH2 were more central in HIV+ AS+ than HIV suggesting their role in regulating miRNA and mRNA expression in persons living with HIV and atherosclerosis. IL13, IL5, TNFβ, and IL1-RA associated with ZBTB7C, were more central in HIV than HIV+ AS+ groups, along with hsa-miR-144-5p, hsa-miR-451a, and hsa-miR-182-5p. hsa-miR-451, one of the miRNA independently validated in this study, has been shown in rats to target the IL6 receptor in arterial smooth muscle cells^110^, and IL-6 knockdown in mice induced release of cytokines including IL6, TNF, CCL5/RANTES, and CCL3/MIP1α^111^. This is corroborated by increased centrality of these downstream cytokines in the HIV+ AS+ group. hsa-miR-182-5p, on the other hand, is a pro-atherosclerotic AKT1-inhibitor implicated in acute coronary disease (atherosclerosis of the heart)^112^ via endothelial cell dysfunction. Positive association between pro-AS miRNAs and anti-inflammatory/anti-AS cytokines such as IL5^113^ might suggest a compensatory response that delays AS. Thus, our network analysis identifies two critical atherosclerosis-related miRNAS− hsa-miR-182-5p which affects endothelial cells, and hsa-miR-144-5p (also validated by RT-qPCR) which affects macrophages, known to be elevated in high viral titer HIV infection.

## Conclusion

Taken together, this study revealed novel miRNAs differentially expressed in AS+ /- people living with HIV, and identified many interesting interactions of miRNAs with cytokines. Centrality measures indicate that miRNAs display importance in regulating molecular networks in people living with HIV, whereas cytokines play a greater role in people living with HIV and AS. Though mRNA measurements were highly variable even with very strict subject selection criteria, novel computational approaches such as single person transcription factor score, and leveraging public domain datasets, provided insights about regulatory factors. Nevertheless, several findings in this study require future validation in larger cohorts with more demographic variables and further expansion of the experimental groups.

## Supplementary Information

Supplementary Information.
