# Supplementary material for: Molecular characterization of atherosclerosis in HIV positive persons
Source: Sci Rep. 2021 Feb 5;11:3232. doi: 10.1038/s41598-021-82429-4 (PMC7865026; doi:10.1038/s41598-021-82429-4)
Supplement: Supplementary file 1 — Supplementary Information. [file 41598_2021_82429_MOESM1_ESM.zip › 41598_2021_82429_MOESM1_ESM.zip/supplemental_11162020/Manuscript info.docx]

**Molecular characterization of atherosclerosis in HIV positive persons**

Adam Cornwell^1^, Rohith Palli^2,3^, Meera V. Singh^4^, Lauren Benoodt^3^, Alicia Tyrell^5,6^, Jun-ichi Abe^7,8^, Giovanni Schifitto^5,6^, Sanjay B. Maggirwar^9^ and Juilee Thakar^4,10,*^

^1^Department of Biomedical Genetics, University of Rochester, Rochester, NY

^2^Medical Scientist Training Program, University of Rochester, Rochester, NY

^3^Biophysics, Structural, and Computational Biology PhD Program, University of Rochester, Rochester, NY

^4^Department of Microbiology and Immunology, University of Rochester, Rochester, NY

^5^Department of Neurology, General Neurology, University of Rochester, Rochester, NY

^6^Department of Imaging Sciences, University of Rochester, Rochester, NY

^7^Department of Cardiology - Research, Division of Internal Medicine, The University of Texas MD Anderson Cancer Center, Houston, TX

^8^Texas A&M Health Science Center Institute of Biosciences and Technology, Houston, TX

^9^Department of Microbiology, Immunology, and Tropical Medicine, George Washing University, Washington, DC

^10^Department of Biostatistics and Computational Biology, University of Rochester, Rochester, NY

*To whom correspondence should be addressed

Juilee Thakar, PhD

601 Elmwood Avenue, BOX 672,

Rochester, NY 14642

Email: juilee_thakar@urmc.rochester.edu
