# Supplementary material for: Molecular characterization of atherosclerosis in HIV positive persons
Source: Sci Rep. 2021 Feb 5;11:3232. doi: 10.1038/s41598-021-82429-4 (PMC7865026; doi:10.1038/s41598-021-82429-4)
Supplement: Supplementary file 1 — Supplementary Information. [file 41598_2021_82429_MOESM1_ESM.zip › 41598_2021_82429_MOESM1_ESM.zip/supplemental_11162020/Supplementary Figure 1.docx]

Supplementary Figure 1: Expression of miRNAs that are differentially expressed between HIV+AS+ vs HIV+AS-, in different blood cell-types from healthy samples. The data is obtained from Juzenas et al.[1].


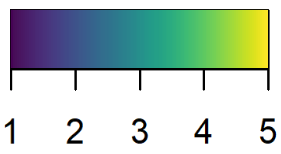

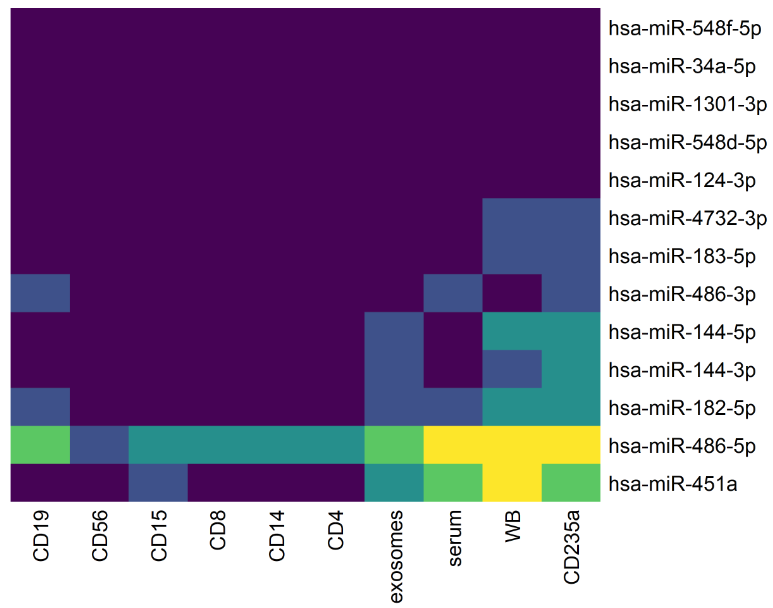


Expression bin

1

3

5

miRNAs

cell types

1. Juzenas, S., et al., *A comprehensive, cell specific microRNA catalogue of human peripheral blood.* Nucleic Acids Res, 2017. **45**(16): p. 9290-9301.
